# Malaria, a difficult diagnosis in a febrile patient with sub-microscopic parasitaemia and polyclonal lymphocyte activation outside the endemic region, in Brazil

**DOI:** 10.1186/1475-2875-12-402

**Published:** 2013-11-07

**Authors:** Patrícia Brasil, Anielle P Costa, Cecilia L Longo, Sidnei da Silva, Maria F Ferreira-da-Cruz, Cláudio Tadeu Daniel-Ribeiro

**Affiliations:** 1 Laboratório de Doenças Febris Agudas, Instituto de Pesquisa Clínica Evandro Chagas (IPEC), Fundação Oswaldo Cruz (Fiocruz), Av. Brasil 4365. Manguinhos, Rio de Janeiro, RJ CEP 21.045-900, Brazil; 2 Serviço de Parasitologia, IPEC, Fiocruz, Av. Brasil 4365. Manguinhos, Rio de Janeiro, RJ CEP 21.040-900, Brazil; 3 Laboratório de Pesquisas em Malária, Instituto Oswaldo Cruz (IOC), Fiocruz, Pavilhão Leônidas Deane - 5° andar, Av. Brasil 4365, Manguinhos, Rio de Janeiro, RJ CEP 21.045-900, Brazil; 4 Centro de Pesquisa, Diagnóstico e Treinamento em Malária (CPD-Mal), Fiocruz, RJ, Brazil

**Keywords:** Atlantic forest, Rio de Janeiro, Fever of unknown origin, Malaria, PCR diagnosis, Polyclonal lymphocyte activation

## Abstract

A case of autochthonous *Plasmodium vivax* malaria with sub-microscopic parasitaemia and polyclonal B-cell activation (PBA) (as reflected by positive IgM and IgG serology for toxoplasmosis, cytomegalovirus, and antinuclear and rheumatoid factors) was diagnosed by polymerase chain reaction (PCR) after consecutive negative rapid diagnostic test results and blood films. The patient, a 44-year-old man from Rio de Janeiro state, Brazil, had visited the Atlantic Forest, a tourist, non-malaria-endemic area where no autochthonous cases of ’bromeliad malaria‘ has ever been described. The characteristic pattern of fever, associated with PBA, was the clue to malaria diagnosis, despite consecutive negative thick blood smears. The study highlights a need for changes in clinical and laboratory diagnostic approaches, namely the incorporation of PCR as part of the current routine malaria diagnostic methods in non-endemic areas.

## Background

Malaria is a potentially fatal disease, especially for individuals exposed for the first time. The chances of death are 108 times higher if a malaria case is diagnosed and treated in non-endemic areas, such as Rio de Janeiro [1], than if it is observed inside the Amazon region, where malaria is concentrated (99.8% of cases) and health personnel are more used to and experienced with the disease diagnosis [2]. The resulting delay in diagnosis is explained not only by limitations in the management experience of non-specialists from non-endemic areas, but also by limited access to experienced microscopists [3].

The non-specificity of clinical presentation of acute malaria makes it even harder to distinguish from other febrile conditions on only clinical grounds, before the spiking pattern of fever shows up or even if it never appears. Thus, a travel history is essential in any febrile patient to evocate malaria diagnosis, mainly in areas of other endemic infectious diseases, such as dengue in Rio de Janeiro, where tropical diseases are not prevalent [4].

Although the extra-Amazonian region is considered a non-endemic area for malaria, autochthonous cases known as the "bromeliad-malaria" are reported in areas inside or near the Atlantic Forest, which provides an excellent environment for *Anopheles (Kerteszia) bellator* and *Anopheles (Kerteszia) cruzii* that can transmit *Plasmodium* and use the water collection in bromeliad to their breeding and larval habitat [5,6]. Autochthonous malaria in the Atlantic Forest (1,300,000 km^2^ distributed over 17 states) are reported mainly in the states of São Paulo [7-10] and Espírito Santo [11], in the Ribeira Valley and Atlantic Coast, and are most often due to *Plasmodium vivax*[7-11]. In the state of Rio de Janeiro, the rare cases of malaria transmission was restricted, until 2010, to a localized area, named Lumiar [3,12,13], a district of Nova Friburgo [14] and a mountainous tourist region, of 700 metres altitude with temperatures between 18°C and 24°C, and 170 km far from Rio de Janeiro [13]. While the state of Sao Paulo registered 229 autochthonous malaria cases in the period of 2003 to 2010 [15], the state of Rio de Janeiro reported 29 cases, most of them from Lumiar [14,16].

This case describes the first authochtonous reported case in a new touristic region, in Mata Atlantica in the state of Rio de Janeiro and emphasizes the difficulty of malaria diagnosis when no history of prior exposure in endemic areas is associated with consecutive negative rapid diagnostic tests (RDTs) and negative blood film examinations for the presence of the parasite, even by an expert team.

Although polymerase chain reaction (PCR) can detect parasites down to a density of 0.01 parasites per microlitre and would, therefore, be very useful in sub-microscopic parasitaemia malaria, it is not usually available in routine laboratory practice [17] and the diagnosis relies primarily on light microscopy of blood film, the gold standard for malaria diagnosis. However, the high degree of suspicion triggered by the characteristic-spiking pattern of fever in a 48-hour cycle, in a patient with 16 days of disease, associated to the practice of routine surveillance of malaria cases in the Atlantic Forest, prompted the tropical specialists to look for a malaria diagnosis by using PCR. During fever investigation, the incidental laboratory findings of positive antinuclear factor (ANF) and rheumatoid factor (RF) testing and serology for toxoplasmosis and cytomegalovirus IgM and IgG antibodies suggested the possibility of nonspecific polyclonal B-cell activation (PBA), substantiating the malaria diagnosis.

## Case report

A 44-year-old male engineer from Rio de Janeiro, Brazil, presented at Instituto de Pesquisa Clínica Evandro Chagas, Fundação Oswaldo Cruz (IPEC, Fiocruz), with a history of persistent fever (39°C) for 16 days: initially continuous during the first week followed by intermittent high febrile peaks every 48 hours over the most recent nine days. The fever was accompanied by severe headache and chills, and followed by sweating. The man also complained of abdominal pain, nausea, myalgia, and arthralgia. Eleven days prior to his first consultation at IPEC, the patient had visited the emergency department of another hospital for these symptoms and for a single episode of gum haemorrhage, attributed to dengue fever that was not confirmed by laboratory tests. Because his symptoms did not resolve, he visited IPEC for two walk-in consultations (on days 16 and 18 of the disease).

During the first consultation, the patient reported a stay at a small farm in Sana, in the Atlantic Forest during a three-week holiday, 23 days before the onset of fever. The Atlantic Forest is a commonly visited touristic region in the *Serra do Mar* mountain range. The reserve of Sana is situated at an altitude of 735 m and is 165 km northeast of Rio de Janeiro. The median temperature in the area ranges from 10°C to 18°C, in the summer and winter, respectively.

The patient had no co-morbid diseases or any history of blood transfusion, tissue/organ transplantation, intravenous drug use, or travel to a malaria-endemic area. He had not been injured by needle sticks, nor lived or recreated near ports or airports. Furthermore, he had not experienced high fevers in the years prior to this event, and he had not taken drugs with anti-malarial activity either prior to or after the onset of the disease. During physical examination, his body temperature was 39°C, his blood pressure was 110/60 mmHg, and his pulse rate was 93 beats per minute. He appeared pale and had hepatomegaly and abdominal pain, but neither his spleen nor any of his lymph nodes were enlarged, and he did not have a cutaneous rash or any obvious clinical manifestations of bleeding.

A routine laboratory tests diagnosis for fever of undeterminated origin was performed: complete blood count, routine blood chemistry determinations, chest radiograph, erythrocyte sedimentation rate, antinuclear antibodies, rheumatoid factor, blood and urine cultures, Cytomegalovirus IgM antibodies, heterophile antibody, Human immunodeficiency virus antibodies, tuberculin skin test, and, considering the presented fever pattern, blood films, RTC and PCR were requested to exclude the possibility of malaria in the individual.

Laboratory test results on admission on the 16^th^ day of disease were as follows: C-reactive protein, 5 mg/dL; haemoglobin, 11.9 g/dL; haematocrit, 35%; white blood cell count, 4,440/L (2,175 neutrophils and 1,864 lymphocytes); platelet count, 258,000/mm^3^; total serum bilirubin, 0.36 mg/dL (direct fraction, 0.11 mg/dL); creatinine, 0.94 mg/dL; alanine aminotransferase (ALT), 45 IU/L; aspartate aminotransferase (AST), 129 IU/L; serum albumin, 3.8 g/dL; urea, 37 mg/dL; creatinine, 0.94 mg/dL; LDH, 115 UI/L; sodium, 144 mmol/L; potassium, 4.4 mmol/L and glucose, 91 md/dL.

Routine blood cultures, microscopic urinalysis, and urine cultures were all negative. Serologic tests for toxoplasmosis and cytomegalovirus were positive for both IgG and IgM antibodies. The test results for ANF and RF were also positive, showing antibody titres of 320 (speckled pattern) and 64, respectively, whereas the test result for anti-HIV was negative. Abdominal ultrasound, echocardiography, and chest radiography results were normal. A microscopic direct blood examination (thick and thin blood films) was performed every 48 hours and assessed by two independent experts (on days 11, 13, and 15 of the disease), and rapid malaria tests (OptiMal®, on days 16 and 18) repeatedly reported negative results. On day 18 of the disease, single PCR tests for *Plasmodium*[18] and *Plasmodium vivax*[19] were positive, confirming the clinical suspicion of malaria.

Treatment with chloroquine and primaquine was initiated, and the patient’s fever resolved 24 hours after the start of treatment. He made a full clinical recovery and the laboratory abnormalities had normalised by the end of the treatment. The PCR tested negative for the first time on the second day and further negative results were reported on days 7, 21, 28, 40, 60, and 100 after treatment initiation, confirming that the treatment was curative. Serologic test results for toxoplasmosis and cytomegalovirus were negative on day 100 after the onset of symptoms, whereas the titres for both RF and ANF were reduced on the eighth day after treatment.

## Consent

Written informed consent to the publication of this case report was obtained from the patient. A copy of the written consent is available for review by the Editor-in-Chief of this journal.

## Discussion

This is the first report of autochthonous, probably ‘bromeliad malaria’ [6] from Atlantic Forest in the touristic area of Sana, 165 km from the capital, in the state of Rio de Janeiro, where routine surveillance should be increased, as cases in the neighbourhood states of São Paulo and Espírito Santo are occasionnally described [7-11].

The essential clue for the diagnosis of malaria in febrile patients is an accurate history of malaria exposure in endemic areas. The patient described here did not visit the Amazon region, which accounts for 99.8% of registered malaria cases in Brazil [2], and had not travelled outside Brazil on any occasion, nor had any previous experience of epidemiological conditions associated with malaria, such as transfusional, nosocomial or airport malaria. Malaria cases from the Atlantic Forest often present with mild, atypical symptoms and very low levels of parasitaemia [9]. Nevertheless, the case reported here presented headache and classic malarial paroxysms of fever spikes, chills and rigours at regular intervals, in addition to hepatomegaly, possibly because of the delay in diagnosis, after two weeks of disease.

The patient’s long-lasting fever made the diagnosis of dengue and leptospirosis unlikely, in the same way that the absence of enlarged lymphonodes or spleen made infectious mononucleosis and lymphoma improbable. Other infectious diseases, such as typhoid fever, Q fever, rickettsial infections, and hepatitis virus, were not considered or tested for because the resolution of fever after the institution of specific therapy provided good evidence to support the presumptive diagnosis of malaria.

General laboratory finds (blood cells count and biochemistry) were normal, except for the raised C-reactive levels, as commonly described in malaria [11]; a slight elevation of AST, (129 IU/L), and a discrete normocromic and normocytic anaemia (HB = 11.9 g/dL Htc = 35%), which is attributable, but not specific, to malaria, might be the result of a long-standing infection. However, in the fourth week of illness, the patient had no evidence of haemolysis, as LDH and bilirubin levels were normal. The absence of thrombocytopaenia, together with normal glycaemia, might represent the benign aspect of ‘bromeliad-malaria’ with low parasitaemia.

In spite of the negative RDTs and negative parasitaemia by direct examination of blood smears after consecutive 48-hour intervals, despite no previous treatment, PCR tests were positive for *Plasmodium* genera and *P. vivax*. Although PCR assays obviously cannot resolve the significant problem of lack of clinical suspicion in the diagnosis of imported malaria [20], it is clinically useful for the diagnosis of patient with suspected malaria despite repeated negative thick-blood-films. The detection of *P. vivax* DNA in the peripheral blood, together with the classic clinical symptoms of malaria, allowed the initiation of anti-malarial treatment.

The lack of effectiveness of the Optimal® test, which is considered insufficient at less than 100 parasites/μL, was not surprising. Commercially-available RDTs do not have a satisfactory sensitivity in detecting *P. vivax*, as already demonstrated by others [21]. A strong caution against the use of this test alone in health facilities around Rio de Janeiro is recommended.

The presence of ANF and RF at titres of 320 and 64, respectively, at the time of diagnosis and the decrease of these titres after treatment initiation suggest that the up-regulation of these auto-antibodies could have been triggered by a state of PBA accompanying the plasmodial infection. The IgM and IgG antibodies indicating cytomegalovirus and toxoplasmosis, which were present at the time of diagnosis, and the absence of these antibodies after treatment also support this premise.

Acute and chronic malaria, in both rodent experimental models [22,23] and natural human disease [24], are, indeed, usually accompanied by a nonspecific PBA that is reflected by a marked increase in the concentration of immunoglobulins (IgM and IgG) and the presence of antibodies against antigens not related to the parasite, including auto-antigens, in the patient’s serum [25]. PBA most likely results from a generalised stimulation of T- and B-lymphocytes by plasmodial antigens endowed with mitogenic properties [26], which are released during the course of infection. For this reason, PBA usually correlates with the level of ongoing parasitaemia [24], which is in turn associated with the severity of malaria symptoms. The patient described here did not have parasitaemia that could be detected by microscopic examination, which appeared to contradict the patient’s extensive PBA, likely reflected by the diversity of the nonspecific immune responses that were triggered. This finding might indicate that, apart from the number of parasites and level of circulating parasite mitogens, other extrinsic and/or individual (genetic) factors can influence the level of PBA following parasite stimulation. Studies that investigate the immune responses to different inocula in animals with different genetic backgrounds may help to elucidate this concept in the future.

In addition to malaria, other infectious diseases, such as African Trypanossomiasis, acute Chagas disease, CMV or EBV infection and leishmaniosis, are also associated with PBA and would have to be excluded by appropriate laboratory tests in other clinical and epidemiological scenarios.

The genetic characterization of the strain of *P. vivax* causing malaria in this case is not in the scope of this paper and was not explored by the authors. It is, however, an important issue because unusual *P. vivax* strains have been reported in other Atlantic forest cases [7,9]. Further studies with detailed information regarding the genetic characteristics of the infecting strain of this and other cases in different areas the Atlantic Forest are currently being undertaken.

## Conclusions

Accurate identification of *P. vivax* malaria may be a difficult challenge outside endemic regions and this type of observation should be primarily reported to clinicians who are apparently prone to look for possible dengue fever infection but might be less likely to consider a diagnosis of malaria.

Medical practitioners should remain aware of the possibility of malaria in patients presenting fever together with polyclonal lymphocyte activation detected by multiple serological positive reactions.

Although expensive and time-consuming, PCR testing for malaria can be a useful and even exclusive diagnostic tool in areas with very low or unusual malaria transmission, where parasitaemia might be undetectable by microscopic examination. The morbidity of malaria, when misdiagnosed and mistreated, outweighs drawbacks in the use of PCR as part of the current routine diagnostic methods in non-endemic areas.

## Competing interests

The authors have declared that they have no competing interests.

## Authors’ contributions

PB attended the patient and described the case, conceived the study and reviewed the manuscript. APC contributed to data collection and the case description and drafted the manuscript. CLL contributed to data collection and the case description. SS performed the microscopic examination. MFFC performed the molecular genetic diagnosis and contributed to the review of the manuscript. CTDR contributed to the conception and review of the manuscript. All authors read and approved the final manuscript.
